# Supplementary material for: Causal Relationship between Aging and Anorexia Nervosa: A White-Matter-Microstructure-Mediated Mendelian Randomization Analysis
Source: Biomedicines. 2024 Aug 16;12(8):1874. doi: 10.3390/biomedicines12081874 (PMC11351342; doi:10.3390/biomedicines12081874)
Supplement: Supplementary file 1 [file biomedicines-12-01874-s001.zip › Supplementary Figure/Figure S4/Singe SNP plot of MR analysis for TL to ubm-b-2111.pdf]

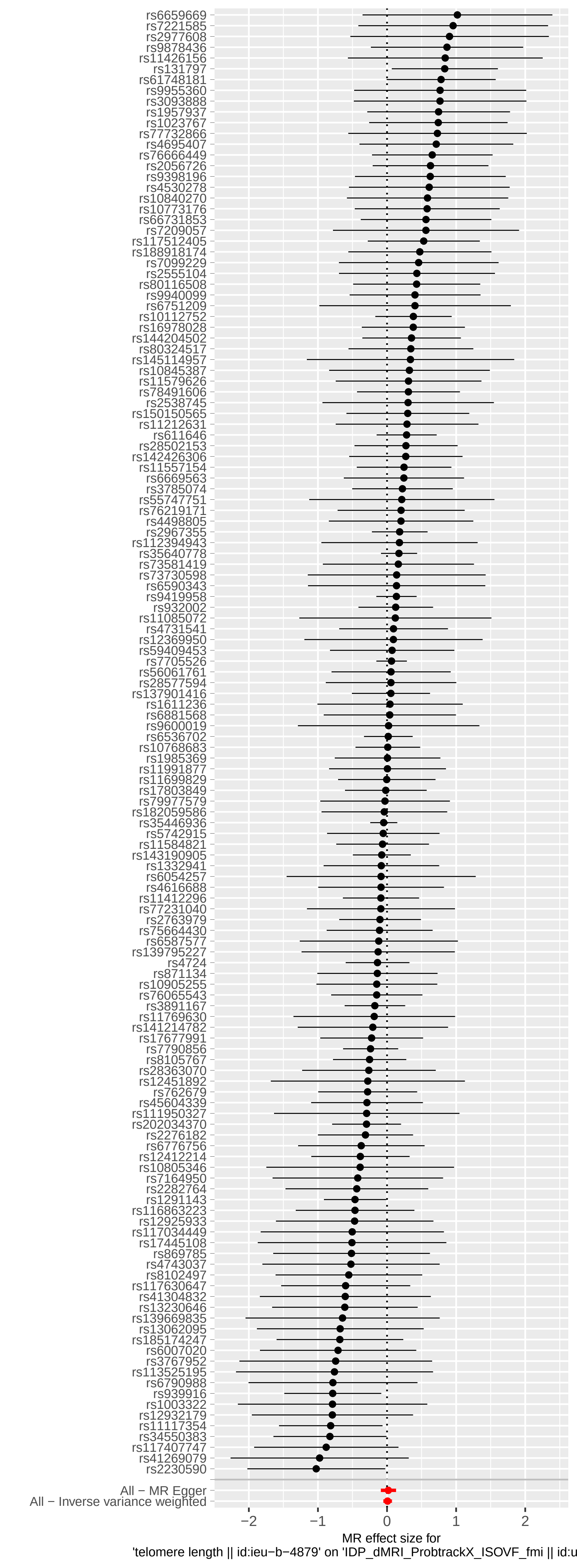

rs6659669
rs7221585
rs2977608
rs9878436
rs11426156
rs131797
rs61748181
rs9955360
rs3093888
rs1957937
rs1023767
rs77732866
rs4695407
rs76666449
rs2056726
rs9398196
rs4530278
rs10840270
rs10773176
rs66731853
rs7209057
rs117512405
rs188918174
rs7099229
rs2555104
rs80116508
rs9940099
rs6751209
rs10112752
rs16978028
rs144204502
rs80324517
rs145114957
rs10845387
rs11579626
rs78491606
rs2538745
rs150150565
rs11212631
rs611646
rs28502153
rs142426306
rs11557154
rs6669563
rs3785074
rs55747751
rs76219171
rs4498805
rs2967355
rs112394943
rs35640778
rs73581419
rs73730598
rs6590343
rs9419958
rs932002
rs11085072
rs4731541
rs12369950
rs59409453
rs7705526
rs56061761
rs28577594
rs137901416
rs1611236
rs6881568
rs9600019
rs6536702
rs10768683
rs1985369
rs11991877
rs11699829
rs17803849
rs79977579
rs182059586
rs35446936
rs5742915
rs11584821
rs143190905
rs1332941
rs6054257
rs4616688
rs11412296
rs77231040
rs2763979
rs75664430
rs6587577
rs139795227
rs4724
rs871134
rs10905255
rs76065543
rs3891167
rs11769630
rs141214782
rs17677991
rs7790856
rs8105767
rs28363070
rs12451892
rs762679
rs45604339
rs111950327
rs202034370
rs2276182
rs6776756
rs12412214
rs10805346
rs7164950
rs2282764
rs1291143
rs116863223
rs12925933
rs117034449
rs17445108
rs869785
rs4743037
rs8102497
rs117630647
rs41304832
rs13230646
rs139669835
rs13062095
rs185174247
rs6007020
rs3767952
rs113525195
rs6790988
rs939916
rs1003322
rs12932179
rs11117354
rs34550383
rs117407747
rs41269079
rs2230590
All − MR Egger
All − Inverse variance weighted
−2
−1
0
1
2
MR effect size for
'telomere length || id:ieu−b−4879' on 'IDP_dMRI_ProbtrackX_ISOVF_fmi || id:u
